# Palliative and end of life care communication as emerging priorities in postgraduate medical education

**Published:** 2016-03-31

**Authors:** Amanda Roze des Ordons, Rola Ajjawi, John Macdonald, Aimee Sarti, Jocelyn Lockyer, Michael Hartwick

**Affiliations:** 1 Department of Critical Care and Division of Palliative Medicine, University of Calgary; 2 Centre for Research in Assessment and Digital Learning, Deakin University, Melbourne, Australia; 3 Division of Critical Care Medicine, University of Ottawa; 4 Department of Anesthesia, University of Ottawa; 5 Division of Palliative Medicine, University of Ottawa; 6 Department of Community Health Sciences, University of Calgary

**Keywords:** Palliative care, End of life care, Mixed methods, Communication skills, Postgraduate medical education

## Abstract

**Background:**

Reliance on surveys and qualitative studies of trainees to guide postgraduate education about palliative and end of life (EOL) communication may lead to gaps in the curriculum. We aimed to develop a deeper understanding of internal medicine trainees’ educational needs for a palliative and EOL communication curriculum and how these needs could be met.

**Methods:**

Mixed methods, including a survey and focus groups with trainees, and interviews with clinical faculty and medical educators, were applied to develop a broader perspective on current experiences and needs for further education. Quantitative descriptive and thematic analyses were conducted.

**Results:**

Surveyed trainees were least confident and least satisfied with teaching in counseling about the emotional impact of emergencies and discussing organ donation. Direct observation with feedback, small group discussion, and viewing videos of personal consultations were perceived as effective, yet infrequently identified as instructional methods. Focus groups and interviews identified goals of care conversations as the highest educational priority, with education adapted to learner needs and accompanied by feedback and concurrent clinical and organizational support.

**Conclusions:**

Our work expands on previous research describing needs for postgraduate education in palliative and EOL communication to include the importance of support, culture change, and faculty development, and provides insight into why such needs exist.

## Introduction

With an aging population, increasing prevalence and complexity of chronic disease, and a limited number of palliative care specialists, Internal Medicine physicians will need to have a greater role in providing palliative care to seriously ill patients in hospital.^1^,^2^ Palliative care has been defined as holistic care that relieves suffering and improves the quality of life for people living with or dying from advanced life-limiting illness, through attention to physical, psychological, social, and spiritual needs of the person and their family.^3^

Surveys of internal medicine trainees describe challenges communicating about palliative and end of life (EOL) care.^4^–^7^ Observational studies have identified that trainees insufficiently address prognosis,^8^ neglect essential steps of shared decision-making,^9^ and often do not seek patient values, concerns and preferences or address emotion,^8^–^10^ resulting in misinterpretation of EOL preferences.^11^ These findings are of particular concern, as therapeutic relationships are founded upon rapport,^12^ and prognostic information significantly impacts decision-making.^13^ Patient-centered communication has been identified as the highest priority competency in the domain of palliative care for internal medicine trainees to achieve prior to graduation.^14^

Prior research identifying needs for communication skills education in palliative and EOL care for internal medicine trainees have adopted either a quantitative^6^,^15^,^16^ or qualitative approach alone^17^,^18^ and limited their scope of inquiry to trainee perceptions.^6^,^15^–^18^ Survey findings are confined by the questions asked and can only speculate on the reasons for identified needs, whereas focus groups and interviews provide depth yet sacrifice breadth of information;^19^ a mixed methods approach can address some of these limitations.

With the principles of curriculum design in mind,^20^ we performed a mixed-methods needs assessment as a first step in informing the development of a communication curriculum for internal medicine trainees. We sought to describe current education in palliative and EOL communication skills within an internal medicine training program, determine the priorities and optimal instructional methods, and explore ideas for development of a future curriculum to advance communication skills in palliative and EOL care.

## Methods

### Study design

We adopted a mixed methods approach, including surveys, focus groups and interviews of trainees, clinical faculty and medical educators. The communication skills focused on were those listed under the 2011 CanMEDS Objectives of Training for Internal Medicine described by the Royal College of Physicians and Surgeons of Canada (RCPSC) that related to palliative and EOL care. These objectives include establishing rapport with patients and their families, informed consent, discussing bad news, advance care planning, goals of care, family meetings to develop a shared plan of care, addressing conflict, discussing organ donation, and counseling about the emotional impact of emergency situations.^21^

### Data collection

Quantitative data were collected through surveys of trainees, and qualitative data through survey free text entries, focus groups with trainees, and semi-structured interviews with clinical faculty and educators from a single Canadian Internal Medicine training program. The investigators developed a survey based on previous literature and the CanMEDS competencies for the communicator role.^22^ The survey was reviewed by a survey methodologist, and pilot-tested with eight trainees to establish face and content validity; the wording of several questions was modified as a result. The survey collected non-identifying demographic data, opinions of the relative importance of communication, current communication teaching, and needs for further teaching (Online supplemental material, Appendix I).

Focus groups were conducted to explore current communication teaching, using a standardized focus group guide developed by the investigators. Focus groups were facilitated by an experienced research assistant and attended by the principal investigator. The principal investigator conducted semi-structured interviews with clinical faculty and educators. The project received approval from the University of Ottawa Research Ethics Board.

### Recruitment, sampling and participants

All trainees in post-graduate years (PGY) 1 – 3 of the core Internal Medicine training program were invited to participate in the survey and focus groups, from May to August 2013 (*n* = 93). Clinical faculty from the Department of Medicine and medical educators with expertise in communication teaching identified through purposive sampling^23^ were invited for an interview.

### Data analysis

Quantitative data were analyzed with SPSS and R software. For questions where respondents were asked to rank perceived importance and confidence in CanMEDS roles, the mean and standard deviation for each role were calculated. Responses from five-point Likert scales were converted to a numerical scale and the median and standard deviation computed. For questions with dichotomous response options, percentages were calculated.

All interviews and focus groups were audio recorded and transcribed. The data were analyzed using thematic analysis,^24^ beginning with listening to audio recordings while reading the transcripts, followed by multiple close readings of each transcript. Two of the investigators (AR, RA) independently analyzed five transcripts to inductively develop preliminary codes. The coders discussed and negotiated the framework and discrepancies to establish consensus. AR then analyzed and indexed all remaining transcripts using constant comparison.^24^ RA examined the quotes assigned to each code to ensure consistency in coding. The data were then charted, mapped and interpreted to develop themes and subthemes.^23^

## Survey Results

### Demographics

The survey response rate was 43% (*n* = 40/93). The mean (*SD*) age of trainees was 28 +/− 2.3 years, with 60% of respondents indicating female and 27.5% male. Of the respondents, 60% were in PGY 1 year, 30% PGY2, and 7.5% PGY3.

### CanMEDS roles

Trainee perceptions of the relative importance and their self-confidence in the CanMEDS roles are indicated in Table 1. The majority of trainees ranked the medical expert role as most important, followed by the communicator role. For self-confidence, the most frequent highest ranking was in the communicator role, followed by collaborator.

### Communication topics

The majority of respondents (*n*=28/37; 76%) perceived that formal teaching in palliative and EOL communication is currently incorporated into their training, with 1 – 10 hours/year of teaching time. There was significant response variability for topics addressed in formal teaching. More than half of trainees had received teaching in delivering bad news, discussing goals of care, informed consent, and establishing rapport. Formal education about leading family meetings, discussing organ donation, or counseling about the emotional impact of emergency situations was uncommon.

Trainee assessment of satisfaction with teaching, self-confidence and topics of future interest is detailed in Table 2. Trainee satisfaction with current communication teaching was topic-dependent. On a scale of 1 to 5 (1 = not at all satisfied; 5 = very satisfied), the mean score was above 3 for all topics except for counseling about the emotional impact of emergency situations and discussing organ donation. Trainee self-confidence in addressing the various communication topics paralleled their satisfaction with teaching. Despite their reported satisfaction and self-confidence, trainees were interested in further learning for all topics, with the least interest in further education about establishing rapport.

### Methods of teaching and learning

Current communication teaching, as reported by trainees, mostly involved instructor-driven large group presentations, along with objective structured clinical examinations (OSCEs), role modeling and informal discussion. Online learning modules and supervised communication in the clinical setting with feedback were less common. Self-reflective writing, assigned readings, video recorded consultations and role play were very uncommon. Table 3 compares current teaching methods with methods trainees perceived as effective. Of note, three of the methods that trainees most valued (direct observation with feedback, small group discussion, and video recorded consultations) were rarely used.

## Qualitative Results

Five focus groups were held with a total of nineteen trainees (PGY1 *n* = 12; PGY2 *n* = 4; PGY3 *n* = 3). Semi-structured interviews were held with clinical faculty (*n* = 8), and medical educators (*n* = 4). Each focus group and interview lasted up to sixty minutes.

Qualitative data were categorized as I) needs for communication teaching in the current context, and II) ideas for education in communicating about palliative and EOL care.

### Description of needs

Qualitative analysis identified four themes reflecting needs for effective education and practice in palliative and EOL communication: trainee education, faculty development, support, and transformation of cultural philosophy (Table 4).

### Trainee education

Educational needs were related to the structure, content, and process of teaching. Participants thought interventions would be most effective if initiated in medical school, and continued longitudinally into postgraduate education and continuing professional development (CPD). Integrating curriculum interventions into formal teaching and within the clinical setting, and aligning instructional methods, objectives, and assessment strategies were desirable.

Content-related learning needs included all of those described in our survey, as well as telephone conversations, autopsy consent and cultural and spiritual considerations. Content needs perceived only by clinical faculty and educators included communication through translators and written communication.

Process-related educational needs included formal training, with large group sessions to provide a cognitive framework, small group discussions, role play, and simulation with debriefing*.* The importance of informal impromptu teaching and learning in the clinical setting was strongly emphasized, including identifying individual learner goals, role modeling, observation by faculty, specific feedback, peer mentorship, and self-reflection. Educators highlighted the need for both formal and informal assessment of communication, such as OSCEs and feedback during clinical rotations.

### Faculty development

Trainees, clinical faculty and educators all identified the need for CPD in educating about communication in palliative and EOL care. Content areas included building rapport, goals of care conversations, conflict management, integrating communication teaching into the clinical setting, and providing effective feedback to trainees about communication skills. Clinical faculty also highlighted the importance of experiential learning strategies in CPD sessions.

### Support

The need to better support trainees, clinical faculty and educators in teaching and learning about communication related to palliative and EOL care was identified. Trainees viewed clinical faculty roles in teaching, assessment, and sharing patient responsibility as obligations, and fulfilling these roles as a sign of respect for and source of comfort to patients, providing credibility and expertise. Trainees expressed that clinical faculty support through coaching and debriefing would provide “in the moment” content expertise for challenging conversations and difficult decisions, while validating trainees’ knowledge and skills. A common need amongst trainees, clinical faculty and educators was multidisciplinary support in facilitating challenging conversations with patients and families*.*

Educators noted the importance of expertise and leadership in developing and implementing a curriculum for palliative and EOL communication. Both educators and clinical faculty identified support from local and national professional organizations as essential for successful curricular implementation.

### Cultural transformation

All participant groups emphasized the need to prioritize education about palliative and EOL communication, identify individual learner goals, and provide specific feedback. Clinical faculty commented that trainees will need to become more comfortable with death and dying to have meaningful conversations with patients and families about palliative and EOL care.

### Ideas for improvement

Trainees, clinical faculty and educators identified strategies for improving education about palliative and EOL communication. Ideas were related to curriculum planning, educational content, assessment strategies, support, and cultural transformation, as summarized in Table 5.

## Discussion

Through a mixed methods approach, the current study provides insight into educational needs for palliative and EOL communication within an internal medicine program. Our research expands on previously reported descriptions of the need for postgraduate communication teaching in palliative and EOL care in Canada.^15^,^17^,^18^ We included both trainees and faculty in our study, whereas previous research focused only on trainees.^15^,^17^,^18^ This enabled us to identify shared perspectives, and provided a richer description of needs than would have been possible had only one group participated in the study. We identified the need for additional support, culture change, and faculty development, findings not described in the previous literature.

### Trainee needs for education in palliative and end of life communication

The need for additional formal and informal education in all assessed areas of palliative and EOL communication was clear, regardless of satisfaction with current teaching and self-perceived confidence. Survey findings were restricted by the response options provided and the response means clustered within a narrow range, affording a limited ability to distinguish perceived priorities for future educational initiatives. These limitations were counterbalanced by the qualitative data, which expanded upon needs to include transformation of the learning environment and support; this data also clarified priorities, and offered insight into the mechanisms contributing to needs. For example, while survey responses suggested that trainees were relatively confident in discussing goals of care and that organ donation was the greatest priority for learning, the focus groups and interviews suggested otherwise, drawing attention to the significant need for corresponding education and mentorship in conversations about goals of care. The importance of ongoing education in EOL conversations was illustrated by narratives colored by uncertainty, anxiety, feelings of abandonment, and moral distress amongst trainees, and past and present challenges recounted by faculty. Despite the previously described survey findings, our qualitative data therefore suggest that communication about goals of care remains a significant challenge with considerable impact on healthcare professionals and their patients.

The discordance between survey results and qualitative findings around goals of care conversations warrants further exploration. It is possible that survey questions did not promote deep reflection and responses reflected a social desirability bias, whereas focus groups and interviews provided context through narrative. Focus group participants perhaps realized they were not alone in their experiences, facilitating more honest self-assessment. Alternatively, the perceived confidence reported in the survey may be related to the frequency of EOL conversations, with experience instilling a false sense of security. Prior research has identified that more experience with EOL care is associated with greater self-perceived competence,^16^,^25^ however self-assessments do not correlate with patient, family, or faculty assessments.^26^ This confidence-performance gap is of concern, considering the reliance on clinical experience as a teacher and acceptance of learning through trial and error.^27^ As unhelpful communication can cause iatrogenic suffering, with a lasting impact upon patients and families^28^ and residual uncertainty and emotional distress amongst trainees,^17^ difficult discussions should be considered *as seriously as an invasive procedure*. Graded supervision until demonstration of competency is indicated to mitigate these potential harms in providing support for trainees and their patients in EOL conversations and decision-making.^7^

Considering the frequency, impact, and physician lack of confidence in discussing goals of care in the acute care setting, there is a considerable need for education and support across the spectrum of medical education. As recognized by trainees in our study, integrating teaching and assessment into the formal curriculum and the clinical setting will be necessary but not sufficient; a change in cultural philosophy within both medicine and society will also be required to provide the context for open conversations and decision-making around palliative and EOL care.

### Faculty education needs

Clinical faculty members are involved in medical education through role modeling and guidance, helping trainees derive contextualized meaning from their experiences. These roles are especially important in communication about palliative and EOL care, with communication being linked to self-concept, and where difficult conversations and decisions can lead to emotional distress amongst trainees, patients, and families.^10^,^17^,^29^ Some clinical faculty in our study lacked confidence in their own communication skills and their ability to provide feedback to trainees. This was related to the absence of prior education in these domains, and impacted trainee education and patient care. Implementing faculty development strategies alongside trainee education will be essential for the success of programs aiming to improve trainee communication skills in palliative and EOL care.

Previous literature has described barriers to providing feedback on communication skills, including fear of eliciting trainee emotional and defensive reactions, time pressures, lack of opportunity for direct observation, and brief trainee-faculty relationships^30^ and concern about personal repercussions.^31^ Furthermore, faculty inexperience in providing feedback has been cited as a rate-limiting step in transitioning to competency-based medical education (CBME).^32^ With increasing focus on CBME and the attendant need for performance assessment in simulated and workplace settings, provision of regular and effective feedback will be expected.^33^ Certainly, skills in providing feedback can be improved.^34^ A systematic review of faculty development initiatives highlighted the importance of multimodal educational strategies involving active participation, applied learning, feedback and peer networks to support maintenance and ongoing learning.^35^ Faculty development initiatives related to communication about palliative and EOL care will need to involve skills-based learning, while addressing attitudinal barriers and contextual challenges.

### A shift in culture

Contemporary Western medicine tends to focus on cure and interventions to avoid death at all costs, an orientation that previous literature has also identified amongst trainees.^18^ Recognizing the limits of what medicine can achieve and that many patients prioritize quality over length of life^36^ may help trainees achieve the empathic and meaning-laden communication that fosters therapeutic relationships and aligns medical care with patient values and goals. Incorporating palliative care into the curriculum early and working with patients at and near the end of life may facilitate such realizations.

### Closing the gap

We have identified a gap in education about palliative and EOL communication – the need for learning situated in relevant clinical experience, with reinforcement and ongoing development over time is currently not being met. The experiences of internal medicine trainees and clinical faculty suggest that education alone will be insufficient to prepare trainees to have conversations about palliative and EOL care. Support and transformation of the learning environment will also be necessary. These concepts can perhaps be presented within a framework of relational learning, where learning is interdependent and contextually situated in relationships with patients, families, colleagues, and other healthcare providers.^37^,^38^ Application of these concepts to education in palliative and EOL communication highlights the value of collaborative models of interprofessional learning, as well as role modeling and peer mentorship across the spectrum of medical education. In reminding us that we are at once teachers and learners, a relationship-centered approach to education in palliative and EOL communication may cultivate the trust needed for effective mentoring and bidirectional feedback.

### Limitations

Study limitations include a small survey sample size from a single Canadian Internal Medicine training program - larger multi-centre studies would provide a broader perspective. The small number of PGY 3 trainees participating in the study may have also limited the breadth of experience within our centre. However, the study was conducted near the end of the academic year and trainees in our program encounter many EOL situations from the beginning of their training. Finally, we did not seek the insights and opinions of nurses, social workers, or patients and families, who may have provided additional valuable perspectives to consider.

### Conclusion

Through a mixed-methods approach, we have identified the need to improve education and support for communication skills related to palliative and EOL care in the acute care setting. Transformation of the learning environment and attention to trainee and faculty education in receiving and providing feedback will also be important in addressing the current gaps. As integrated models of communication teaching and learning are developed, the role of interprofessional and peer coaching and mentoring in developing skillful communication across various contexts warrants further study.

## Figures and Tables

**Table 1:** Trainee rankings* of importance of and self-confidence in CANMEDS roles

	Medical expert mean (SD)	Communicator mean (SD)	Collaborator mean (SD)	Professional mean (SD)	Health advocate mean (SD)	Manager mean (SD)	Scholar mean (SD)
Importance	6.2 (1.2)	5.9 (1.0)	3.8 (1.3)	3.6 (1.7)	3.1 (1.6)	2.8 (1.6)	2.6 (1.9)
Self-confidence	3.5 (1.4)	5.9 (1.5)	5.3 (1.6)	5.3 (1.3)	3.3 (1.7)	2.5 (1.4)	2.2 (1.5)

* 1 = least important/least self-confident; 7 = most important/most self-confident

**Table 2:** Trainee satisfaction, self-confidence and interest* in further education in communication topics

Topic	Level of satisfaction with training mean (SD)	Level of self-confidence mean (SD)	Level of interest in further training mean (SD)
Establishing rapport	3.7 (0.5)	4.2 (0.6)	3.2 (0.9)
Informed consent	3.6 (0.7)	4.0 (0.6)	3.6 (0.8)
Adverse event disclosure	3.4 (0.8)	3.2 (1.1)	3.8 (0.8)
Bad news	3.3 (0.9)	3.7 (0.7)	3.4 (0.9)
Advance care planning	3.1 (0.9)	3.2 (0.8)	3.9 (0.8)
Goals of care	3.1 (0.9)	3.5 (0.9)	3.9 (0.9)
Family meetings	3.1 (0.8)	3.3 (1.0)	3.8 (1.0)
Conflict management	3.1 (0.9)	3.0 (1.1)	3.6 (1.0)
Counseling about emotional impact of emergency situations	2.7 (0.8)	2.8 (0.7)	3.7 (0.7)
Organ donation	2.6 (0.9)	2.6 (0.9)	3.7 (0.8)

* 1 = not at all satisfied/confident/interested; 5 = very satisfied/confident/interested

**Table 3:** Current methods of communication skills teaching compared to perceived effective methods

	Methods perceived as effective for initial learning (>50%)	Perceived ineffective methods for initial learning (<50%)
Common current methods of learning (>50%)	Instructor presentations	Large group discussion
	Video demonstrations	
	OSCE/simulation	
	Role modeling/informal discussion	
Uncommon current methods of learning (<50%)	Observation by preceptor with feedback	Role play
	Small group case-based learning	Online learning modules
	Watching video of own consultation	Assigned readings
		Self-reflective writing

% = percentage of respondents

**Table 4:** Needs for communication teaching with examples and illustrative quotes

Themes and subthemes	Examples	Illustrative quotes
***Education – trainees and clinical faculty***		
Structure	Prioritization, timing, longitudinal, multimodal, integrated, alignment, collaboration	*Definitely at the beginning, the very beginning of med school is important (Trainee, PGY3)*
		*Communication to me is iterative, there’s no ceiling effect. You have to continually hone and develop it like any clinical skill in medicine. (Medical educator)*
Process	Formal – cognitive framework, small group discussion, role play, simulation, assessment Informal – role modeling, direct observation and feedback (clinical faculty, nursing, social work), mentorship (peer, clinical faculty), 360 assessments	*Small group… I’m the patient, you’re the resident, and these people are there watching you. And then you can say “Staff, can you offer some advice? What do you think went well?” (Trainee, PGY1)*
		*I think it would be more beneficial if staff came when we have these discussions, let us lead them and then give us feedback right away, on that patient, on that day. (Trainee, PGY2)*
		*Even group reflection. People inevitably will be able to bring out their own experiences or challenges and maybe then it helps people work through issues, discuss, offer advice between each other. (Trainee, PGY1)*
Content	Building rapport, informed consent, discussing bad news, family meetings, conflict management, counseling, prognostication, goals of care, death and dying, organ/tissue donation, autopsy, religion and cultural awareness, working with translators, written communication, teaching and assessment of communication	*I think especially ‘code status’ because that’s a discussion that we do often and I don’t think… I don’t think we do it well. (Trainee, PGY3)*
		*Establishing end of life care plans are also important and can be done very poorly unfortunately. The general public perception of what a DNR is, for example, or what a will is, is very different from reality. We don’t do a great job in communicating that. (Clinical faculty)*
Faculty development	Communication	*I just had an attending whose communication skills are atrocious and I have no idea how it’s gotten so far. I know he’s a bright guy, but it’s also like, “Wow, you could learn what I’ve been learning.” (Trainee, PGY2)*
	Providing feedback	*It would be helpful to have faculty development on how to observe communication and how to give constructive feedback to trainees. (Educator)*
***Support***		
Clinical	Clinical faculty support for residents, peer support, multidisciplinary team support	*Even if a staff said to me, “You’re delivering bad news today. Do you want to talk about it for a few minutes before you do it?” If they sat down and said, “Have you had any barriers? What are they? Let’s take five minutes, let’s share a coffee, let’s talk about it.” That would make my experience so much stronger. (Trainee, PGY1)*
		*It really depends on who’s there to support you… If you’ve got a good team it makes a difficult family meeting easier. (Educator)*
Organizational	Educational leadership, departmental support and RCPSC support for communication teaching	*Even just having someone that’s an expert in the content to sort of take the lead. We all know that we need to teach it better. We need to evaluate it better. It’s just the knowhow. (Medical educator)*
		*So you know, if we value it, then we support it; not just in words, but we support it financially as well. I think that’s a matter for chairs of departments. (Medical educator)*
***Transformation of learning environment***		
Prioritization	Education about palliative and EOL communication	*Trainees can see the disconnect between what I teach formally and then what happens in real life. So I may decide that communication is very important and I’m teaching it to you in a one day workshop, but if I never assess it at the bedside and I don’t talk to you about it, and I never assess it, what does that tell you about the importance of communication skills? (Medical educator)*
Individualization	Adapting educational strategies to learner needs	*I was trying to comply with something that was unnatural to me because I was instilled with a set of rules that I felt I had to follow. (Trainee, PGY1)*
Changing current perspectives	Reframing notions of death and dying	*So the issues around death for me is really something that’s come up a lot and something that I don’t feel comfortable with. (Trainee, PGY1)*
	Assessment *for* learning	*You sign up to be a doctor and you didn’t think you were going to be dealing with death and dying in a population that is aging every minute? But truly they didn’t, so I try to get them comfortable with death. (Clinical faculty)*

**Table 5:** Ideas for improving education about palliative and end of life communication across the continuum of medical education

Domain	Strategy
Transforming the learning environment *“Viewing communication skills with the same value as maintaining our skill sets in critical thinking and in physical examination” – Medical educator*	Prioritizing teaching and learning about communication in palliative and EOL care
	Identifying and adapting the content and teaching methods to learner needs
	Establishing a culture of assessment *for* learning with open and honest bidirectional feedback based on observed behaviours
Support in communicating about palliative and EOL care *“One major message that needs to be conveyed to those above us is that we need you actively involved, not ticking off [checklist] boxes. I think if they have to check off anything it’s, ‘I have spent 20 minutes with this student specifically doing this,” versus, “Communication, check.’” – Trainee, PGY1*	Champions with expertise in palliative and EOL communication and medical education to lead curricular development, guide learners, generate enthusiasm, and provide support
	Helping learners to develop an individualized learning plan
	Peer support through discussion and coaching
	Support from members of the interprofessional healthcare team^*^ through pre-briefing in advance of meetings, presence and input during meetings, and debriefing after meetings with patients and their families
Curriculum planning *“It would be nice to have a three year curriculum where the first year trainees start very basic, and then each year you build on that” – Medical educator*	Early initiation of teaching and learning (eg: medical school, first year postgraduate training)
	Longitudinal teaching and learning with progression of complexity from beginning to end of career
	Alignment of learning objectives with methods of teaching and assessment
	Collaboration between internal medicine programs at a national level and between interprofessional groups (eg: physicians, nursing, social workers, psychologists, spiritual care workers) in curriculum development and delivery with sharing of resources and ideas
	Development of a standardized curriculum for palliative and EOL communication specific to the needs of trainees and clinical faculty in Internal Medicine
	Integrating palliative and EOL communication into formal teaching events
	Integrating palliative and EOL communication into clinical rotations and clinical practice
Content for teaching and learning *“Especially ‘code status’ because that’s a discussion that we have often and I don’t think… I don’t think we do it well” – Trainee, PGY3*	General communication including building rapport, family meetings, conflict management, and working with translators
	Palliative and EOL communication including difficult news, advance care planning, goals of care, emotional counseling, organ and tissue donation, autopsy consent, cultural and religious perspectives on death and dying
	Observation of communication skills related to palliative and EOL care
	Providing feedback about communication skills related to palliative and EOL care

## Appendix 1 Survey of Resident Communication Teaching and Learning

**Instructions:** *Please answer the following questions to the best of your ability. The scoring system for each question varies, please read the description of scoring carefully prior to selecting your answer.*

### I. CanMEDS roles

1. Indicate your opinion on the **relative importance** of the following CanMEDS roles by ranking them from 1 to 7, where 1 is the most important and 7 is the least important. Please do not assign the same number to more than one role.
  **Table:**
  CanMEDS role	Importance
  Medical expert	
  Communicator	
  Collaborator	
  Manager	
  Health advocate	
  Scholar	
  Professional	
2. Indicate your level of confidence in carrying out the following CanMEDS roles by ranking them from 1 to 7, where 1 = most confident and 7 = least confident. Please do not assign the same number to more than one role.
  **Table:**
  CanMEDS role	Level of confidence
  Medical expert	
  Communicator	
  Collaborator	
  Manager	
  Health advocate	
  Scholar	
  Professional	

### II. Current communication training

1. My residency program includes formal/structured training related to the Communicator role
  - Yes
  - No (proceed to section II)
2. Indicate the number of hours per year dedicated to formal/structured training related to the Communicator role in your residency program
  - 1–5 hours
  - 6–10 hours
  - 11–15 hours
  - 16–20 hours
  - >20 hours
3. Indicate whether your residency program provides formal/structured communication training in the following topics
  **Table:**
  Topic	Yes	No
  Establishing rapport/therapeutic relationship		
  Obtaining informed consent		
  Disclosing an adverse event		
  Delivering bad news		
  Advance care planning		
  Decision making concerning resuscitation/‘code status’		
  Holding a family meeting to develop a plan of care		
  Addressing conflict		
  Discussing requests for organ donation		
  Counseling about the emotional/psychological impact of emergency situations		
  Other (please specify):		
4. Indicate your degree of satisfaction with the communication training provided by your residency program for the following topics
  **Table:**
  Topic	Very dissatisfied	Dissatisfied	Neutral	Satisfied	Very satisfied
  Establishing rapport/therapeutic relationship					
  Obtaining informed consent					
  Disclosing an adverse event					
  Delivering bad news					
  Advance care planning					
  Decision making concerning resuscitation/‘code status’					
  Holding a family meeting to develop a plan of care					
  Addressing conflict					
  Discussing requests for organ donation					
  Counseling about the emotional/psychological impact of emergency situations					
  Other (please specify):					
5. Indicate which of the following learning methods are used by your residency program for communication training
  **Table:**
  Learning method	Yes	No
  Assigned readings		
  Online learning modules		
  Watching videos		
  Instructor presentations		
  Large group discussions		
  Small group case-based learning		
  Role play		
  Practice with standardized patients/OSCE		
  Watching a video of own consultation		
  Supervised clinical practice with feedback		
  Mentorship (role modeling, informal discussion)		
  Self-reflective writing (journaling)		
  Other (please specify):		

### II. Needs for additional communication training

1. Indicate your level of confidence in addressing the following topics when communicating with patients and their families
  **Table:**
  Topic	Very uncomfortable	Uncomfortable	Neutral	Comfortable	Very comfortable
  Establishing rapport/therapeutic relationship					
  Obtaining informed consent					
  Disclosing an adverse event					
  Delivering bad news					
  Advance care planning					
  Decision making concerning resuscitation/‘code status’					
  Holding a family meeting to develop a plan of care					
  Addressing conflict					
  Discussing requests for organ donation					
  Counseling about the emotional/psychological impact of emergency situations					

### III. Topics of interest for further communication training

1. Indicate your level of interest in the following topics for communication training
  **Table:**
  Topic	Not at all interested	Uninterested	Neutral	Interested	Very interested
  Establishing rapport/therapeutic relationship					
  Obtaining informed consent					
  Disclosing an adverse event					
  Delivering bad news					
  Advance care planning					
  Decision making concerning resuscitation/‘code status’					
  Holding a family meeting to develop a plan of care					
  Addressing conflict					
  Discussing requests for organ donation					
  Counseling about the emotional/psychological impact of emergency situations					
  Other (please specify):					

### IV. Preferred learning methods for communication training

1. Indicate the degree to which the following methods of learning would be effective for you during ***initial*** communication training
  **Table:**
  Topic	Very ineffective	Ineffective	Neutral	Effective	Very effective
  Assigned readings					
  Online learning modules					
  Watching videos					
  Instructor presentations					
  Large group discussions					
  Small group case-based learning					
  Role play					
  Practice with standardized patients/OSCE					
  Watching a video of own consultation					
  Supervised clinical practice with feedback					
  Mentorship (role modeling, informal discussion)					
  Self-reflective writing (journaling)					
  Other (please specify):					
2. Indicate which strategies would be effective for you in ***preventing a decline*** in communication skills following initial training?
  **Table:**
  Topic	Very ineffective	Ineffective	Neutral	Effective	Very effective
  Assigned readings					
  Online learning modules					
  Watching videos					
  Instructor presentations					
  Large group discussions					
  Small group case-based learning					
  Role play					
  Practice with standardized patients/OSCE					
  Watching a video of own consultation					
  Supervised clinical practice with feedback					
  Mentorship (role modeling, informal discussion)					
  Self-reflective writing (journaling)					
  Other (please specify):					

### V. Barriers to communication training

1. Please indicate the extent to which the following factors interfere with developing communication skills during clinical rotations
  **Table:**
  Factors	Very much interferes	Interferes	Neutral	Does not interfere	Does not at all interfere
  Personal lack of confidence					
  Difficulty transferring skills learned in lectures to clinical practice					
  Difficulty transferring skills learned in simulation to clinical practice					
  Clinical preceptors do not provide good role modeling of communication skills					
  Clinical preceptors seem unprepared to teach communication skills					
  Clinical preceptors presume resident competence in communication skills					
  Time pressures					
  Environmental setting (space, lighting, etc)					
  Other (please specify):					

### VI. Demographics

1. Please indicate your age: _______
2. Please indicate your gender
  - Male
  - Female
3. Please indicate your year of residency training: PGY ______

### VII. Please include your thoughts about a memorable or challenging experience around communication that you have experienced

________________________________________________________________________

________________________________________________________________________

________________________________________________________________________

________________________________________________________________________

________________________________________________________________________

________________________________________________________________________

________________________________________________________________________

________________________________________________________________________

________________________________________________________________________

________________________________________________________________________

________________________________________________________________________

________________________________________________________________________

________________________________________________________________________

________________________________________________________________________

________________________________________________________________________

________________________________________________________________________

________________________________________________________________________

________________________________________________________________________

________________________________________________________________________

________________________________________________________________________

________________________________________________________________________

________________________________________________________________________

________________________________________________________________________
